# Supplementary material for: A hemimetabolous wing development suggests the wing origin from lateral tergum of a wingless ancestor
Source: Nat Commun. 2022 Feb 21;13:979. doi: 10.1038/s41467-022-28624-x (PMC8861169; doi:10.1038/s41467-022-28624-x)
Supplement: Supplementary file 1 — Supplementary information [file 41467_2022_28624_MOESM1_ESM.pdf]

Extended Data for

A hemimetabolous wing development suggests the wing origin from lateral tergum of a wingless ancestor

Takahiro Ohde, Taro Mito and Teruyuki Niimi

Correspondence to: ohde.takahiro.4n@kyoto-u.ac.jp

This PDF file includes:

Supplementary Figures 1 to 13

Supplementary Tables 1 to 4

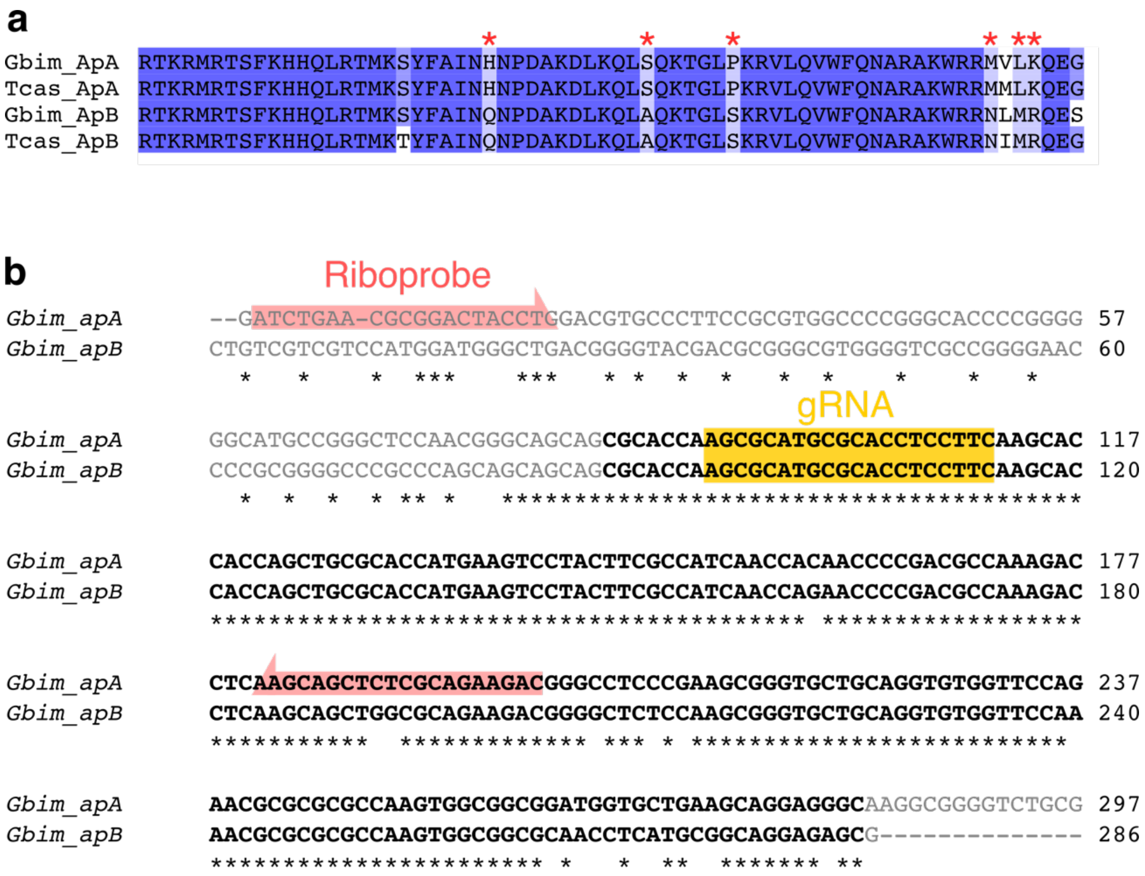

Supplementary Figure1 *ap* paralogues in *Gryllus*

a The multiple alignment of the Ap homeodomains from *Gryllus bimaculatus* (Gbim) and *Tribolium castaneum* (Tcas). Asterisks indicate amino acid residues unique to either ApA or ApB. **b** A partial pairwise alignment of *apA* and *apB* identified in this study. Asterisks indicate matched nucleotides. Bold black text indicates homeodomain sequences. The half arrow and boxes in different colors indicate sequences of PCR primers for synthesizing the riboprobe and design of gRNA, respectively.

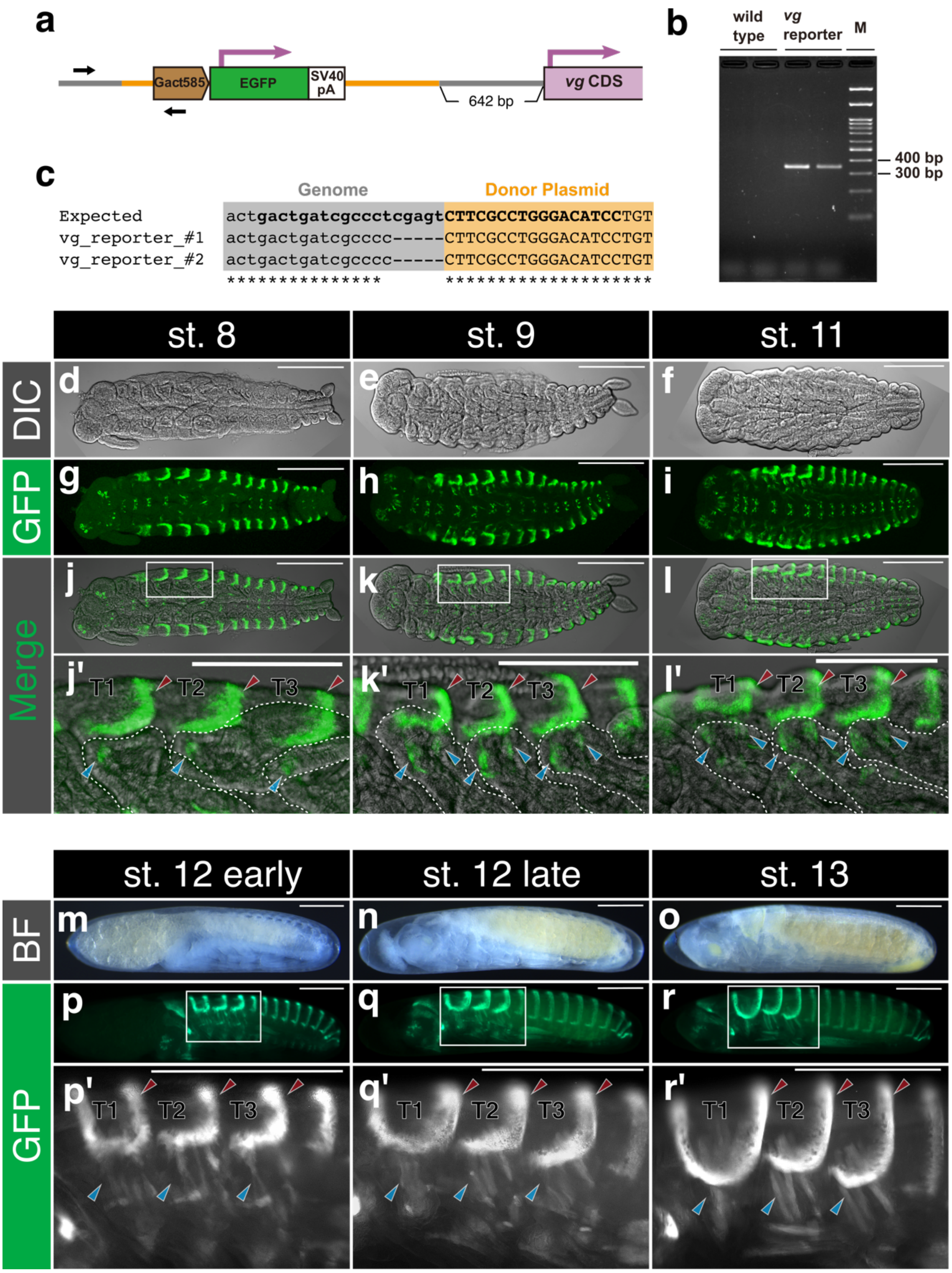
a
Gact585
EGFP
SV40 pA
642 bp
vg CDS
b
wild type
vg reporter
M
400 bp
300 bp
c
Genome
Donor Plasmid
Expected actgactgatcgccctcgagtCTTCGCCTGGGACATCCTGT
vg_reporter_#1 actgactgatcgcccc-----CTTCGCCTGGGACATCCTGT
vg_reporter_#2 actgactgatcgcccc-----CTTCGCCTGGGACATCCTGT
st. 8
st. 9
st. 11
DIC
GFP
Merge
d
e
f
g
h
i
j
k
l
j'
k'
l'
T1
T2
T3
st. 12 early
st. 12 late
st. 13
BF
GFP
m
n
o
p
q
r
p'
q'
r'

Supplementary Figure 2 *vg* reporter gene expression during embryogenesis

a Schematic illustration of the upstream *vg* coding sequence (CDS) with the EGFP reporter cassette. Gray and orange indicate genome and donor plasmid sequences, respectively. Line length is not scaled to sequence length. Black arrows indicate the PCR primer pair used for confirming the insertion. **b** A gel image showing genomic PCR products in the *vg* reporter line. The image shows representative results of two of eight independent samples from each strain. M, DNA size marker. **c** DNA sequences around the boundary between the *Gryllus* genome (lowercase) and the inserted donor plasmid (uppercase). “Expected” shows the expected sequence when genome editing was designed. Bold texts indicate sequences targeted by gRNAs. **d**–**l** Confocal images of vg5´GFP embryos. Maximum projections of confocal stacks are displayed. Differential interference contrast (DIC) microscopy images (**d**–**f**) overlaid with GFP signals (**g**–**i**) are shown in **j**–**l**. **m**–**r** Epifluorescence microscopy images of vg5´GFP embryos from a lateral view. Brightfield (BF) images (**m**–**o**) and GFP signals (**p**–**r**) are shown. Boxed areas in **j**–**l** and **p**–**r** are magnified in **j’**–**l’** and **p’**–**r’**, respectively. Images in **d**–**r’** show representative images of at least three independent embryo specimens. Scale bars are 500 *µ*m and 250 *µ*m in **d**–**r** and **p’**–**r’**, and **j’**–**l’**, respectively.

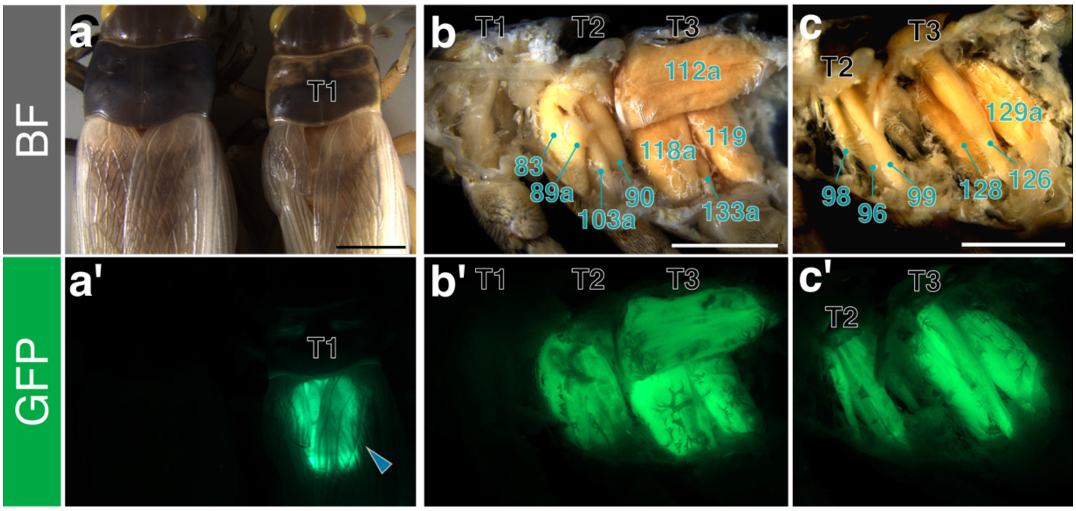

Supplementary Figure 3 *vg* reporter gene expression in adult stage

a, **a'** Dorsal view of vg5′GFP strain (right) compared to wildtype (left). Crickets before cuticle coloration are shown. Arrowhead indicates GFP signal in thoracic muscles. **b**, **b'** Dorsal longitudinal muscle (112a) and dorsoventral muscles (the other numbered muscles) in median section. **c**, **c'** Pleural muscles (all numbered muscles) after removal of medial muscles. Only GFP-positive muscles are annotated, according to Furukawa *et al.* (1983)[1]. Scale bar is 3 mm.

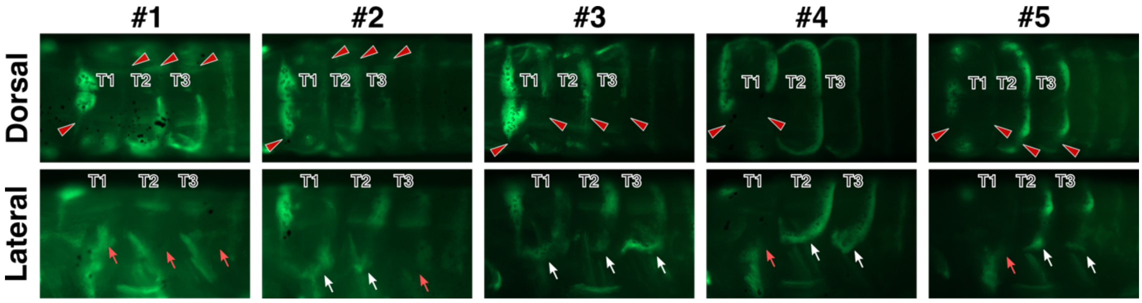

Supplementary Figure 4 ***vg*** **reporter gene expression in** ***apAB*** **crispants**

Stage 13 embryos of vg5´GFP strain after *apAB* sgRNA/Cas9 injection. Five individuals are shown from dorsal and lateral views. Arrowheads indicate loss of reporter expression in tergal margins. Arrows indicate lateral edges of terga. White and red arrows indicate remaining and lost reporter expression at the edges, respectively.

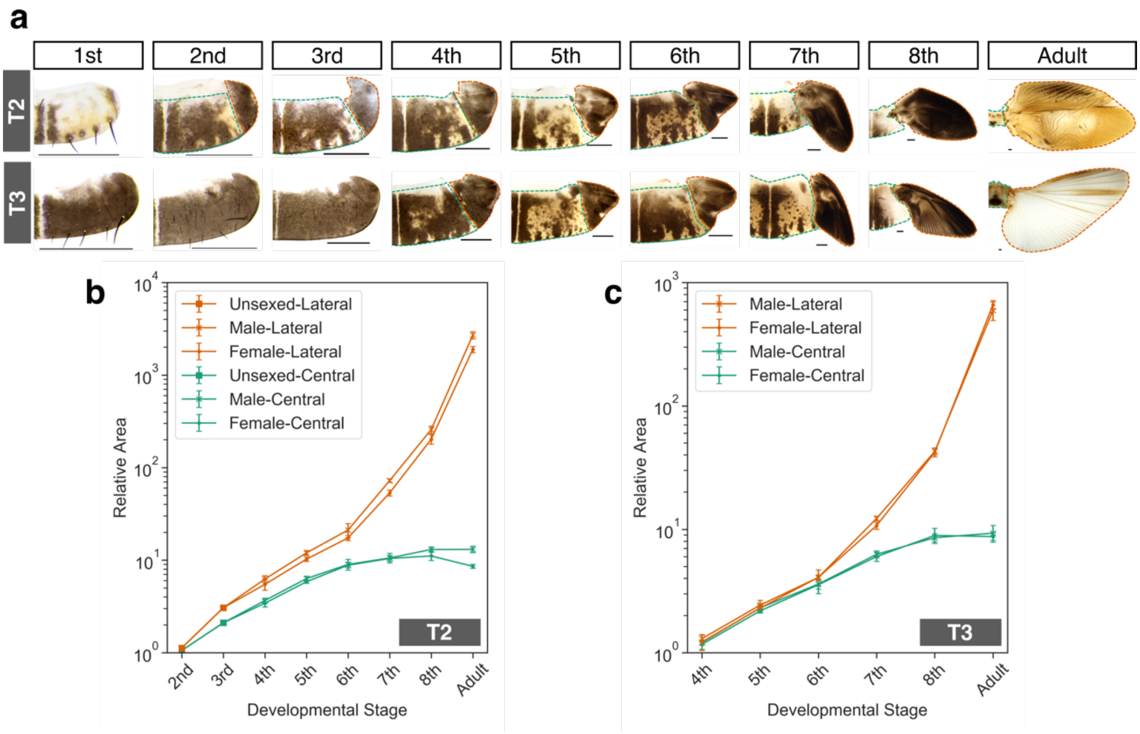

Supplementary Figure 5 Exponential growth of lateral terga

a Representative image of right halves of terga in mesothorax (T2) and metathorax (T3) from first instar nymph to adult. Unsexed and male terga from first to third instar nymphs and from fourth instar nymph to adult are displayed, respectively. Anterior is up. Orange and green dashed lines outline the lateral and central areas, respectively. Scale bar is 0.5 mm. **b**, **c** Size of lateral and central regions in T2 (**b**) and T3 (**c**) during post-embryonic development. The smallest area in each data series is set to 1, and relative areas are plotted for the rest. Mean and standard deviation of n=7 and n=6 biologically independent samples are shown for adult male and the rest of samples, respectively. Source data are provided as a Source Data file.

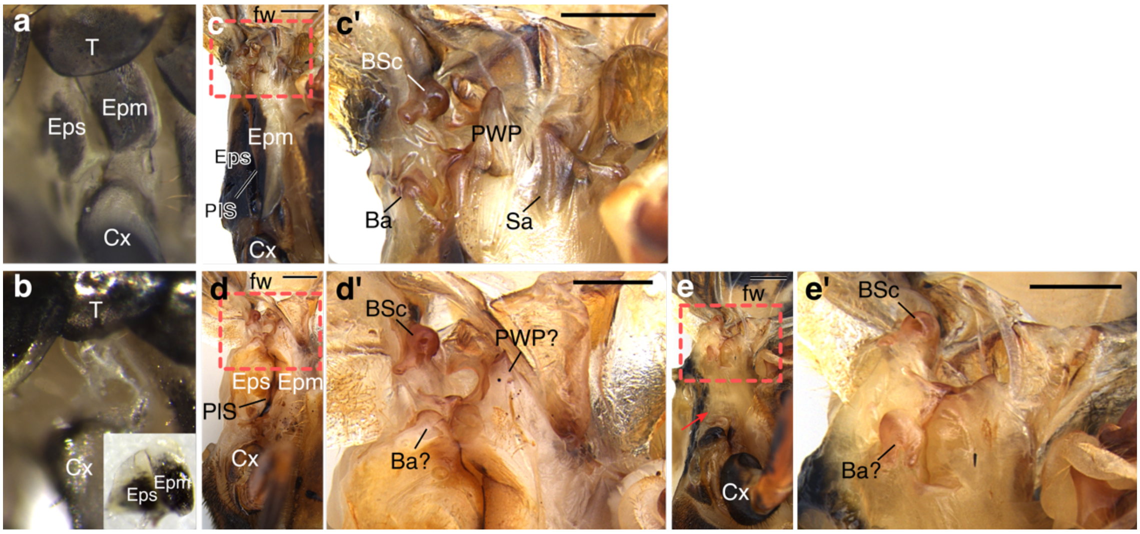

Supplementary Figure 6 Effects of pleural tissue ablation

a, **b** Mesothoracic segments of control (**a**) and treatment (**b**) third instar nymphs from lateral views. Ablated tissue is shown in inset. **c**–**e** Mesothoracic segments of control and treatment adults. Boxed regions are magnified in **c'**–**e'**. Two individuals are displayed for treatment (**d**, **e**). Red arrow in **e** indicates lack of both episternum and epimeron. Scale bar is 1 mm. Ba, basalare; BSc, basicostale; Cx, coxa; Eps, episternum; Epm, epimeron; fw, forewing; PlS, pleural suture; PWP, pleural wing process; Sa, subalare; T, tergum.

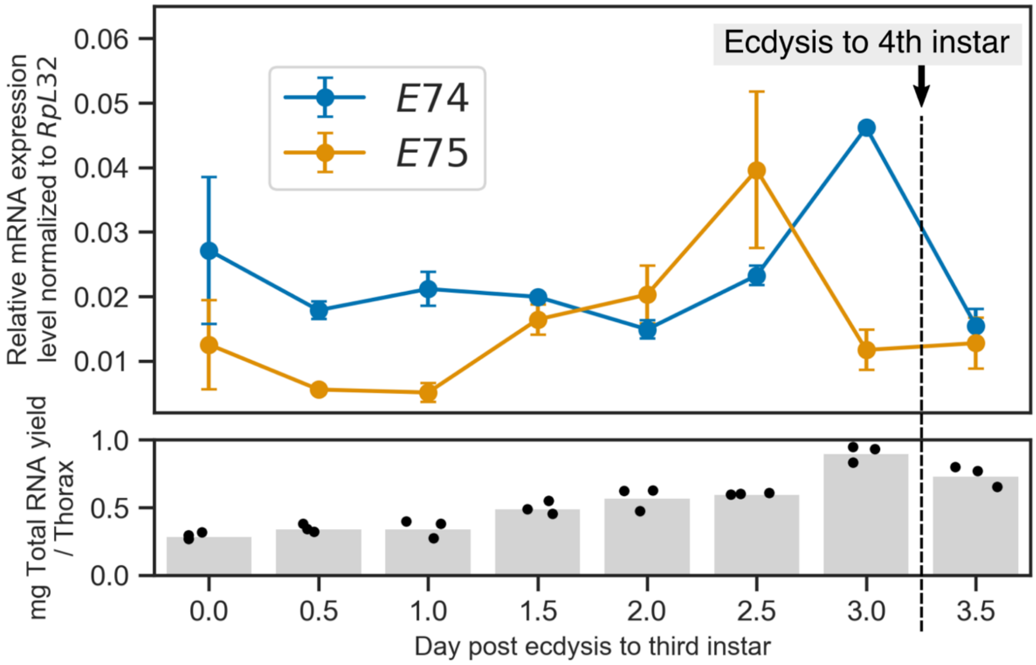

Supplementary Figure 7 Expression level of *E74* and *E75*, and total RNA abundances during third instar nymph

Mean and standard deviation of n=3 biologically independent samples are shown in the upper panel. Mean and value of each sample are shown in the lower panel.

a

SampleID	No. of raw read	Q30(%)	Reads after cutadapt filtering (%reads after cutadapt / raw reads)	Summary of quantification with Salmon		
				num_processed	num_mapped	percent_mapped
d0L1	23,432,749	96.38	23,288,008 (99.4)	23,288,008	22,695,406	97.46
d0L2	23,467,516	96.09	23,374,833 (99.6)	23,374,833	22,626,098	96.80
d0L3	26,132,970	96.26	26,019,161 (99.6)	26,019,161	25,088,571	96.42
d0C1	25,329,751	96.41	25,192,068 (99.5)	25,192,068	24,583,786	97.59
d0C2	19,455,834	96.27	19,372,273 (99.6)	19,372,273	18,834,042	97.22
d0C3	19,568,996	96.44	19,499,781 (99.6)	19,499,781	18,967,121	97.27
d3L1	20,301,543	96.01	20,234,065 (99.7)	20,234,065	19,682,681	97.27
d3L2	27,346,903	95.87	27,247,800 (99.6)	27,247,800	26,435,127	97.02
d3L3	21,023,008	96.03	20,936,611 (99.6)	20,936,611	20,361,017	97.25
d3C1	25,952,754	96.18	25,890,802 (99.8)	25,890,802	25,177,544	97.25
d3C2	21,088,923	96.06	20,977,352 (99.5)	20,977,352	20,434,356	97.41
d3C3	25,111,120	96.15	24,972,627 (99.4)	24,972,627	24,293,625	97.28

b

Total trinity 'genes'	277,660
Total trinity transcripts	356,490
Contig N50 (All)	3,126
Contig N50 (longest)	874
Total assembled base (all)	368,966,335
Total assembled base (longest)	170,990,352

c

n	1,367
Complete and single-copy (%)	31.5
Complete and duplicated (%)	64.1
Fragmented (%)	0.8
Missing (%)	3.6

Supplementary Figure 8 Summary of raw reads, read quantification and de novo assembly
a Summary of raw reads and read quantification with Salmon. **b** Summary of the assembled transcriptome. **c** Summary of transcriptome quality assessed by BUSCO.

a

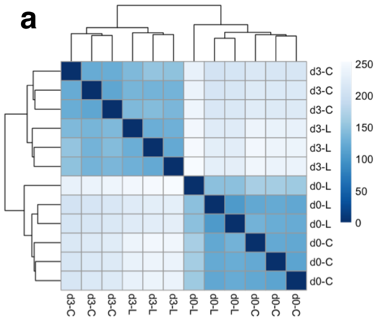

b

Comparison between		*padj* < 0.1	*padj* < 0.05	*padj* < 0.01
Regions -- Lateral vs Central	Lateral	1164	938	652
	Central	1165	866	469
Time points -- 0 DPE vs 3 DPE	0 DPE	3870	3512	2839
	3 DPE	3932	3531	2857

c

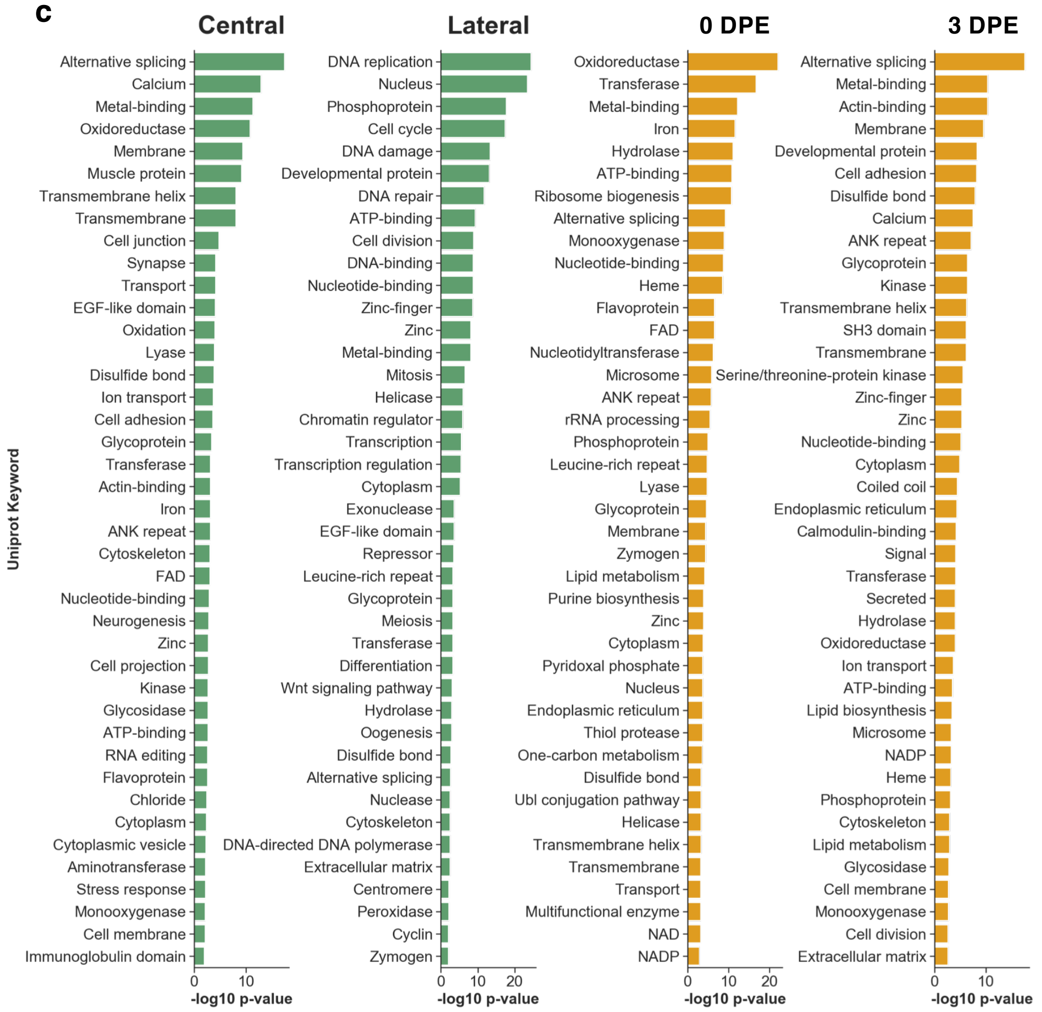

Supplementary Figure 9 Summary of differentially expressed genes (DEG)

a Heatmap of sample-to-sample distance and hierarchical clustering. Color code indicates Euclidean distance. **b** Number of upregulated DEG in each comparison. Numbers in different FDR (*padj*) cut-off are shown. A Wald test was used for significance testing and *P* values were adjusted using the Benjamini and Hocheberg method according to DESeq2 software. **c** Top 40 statistically enriched uniport keyword in transcripts upregulated in each group according to DAVID.

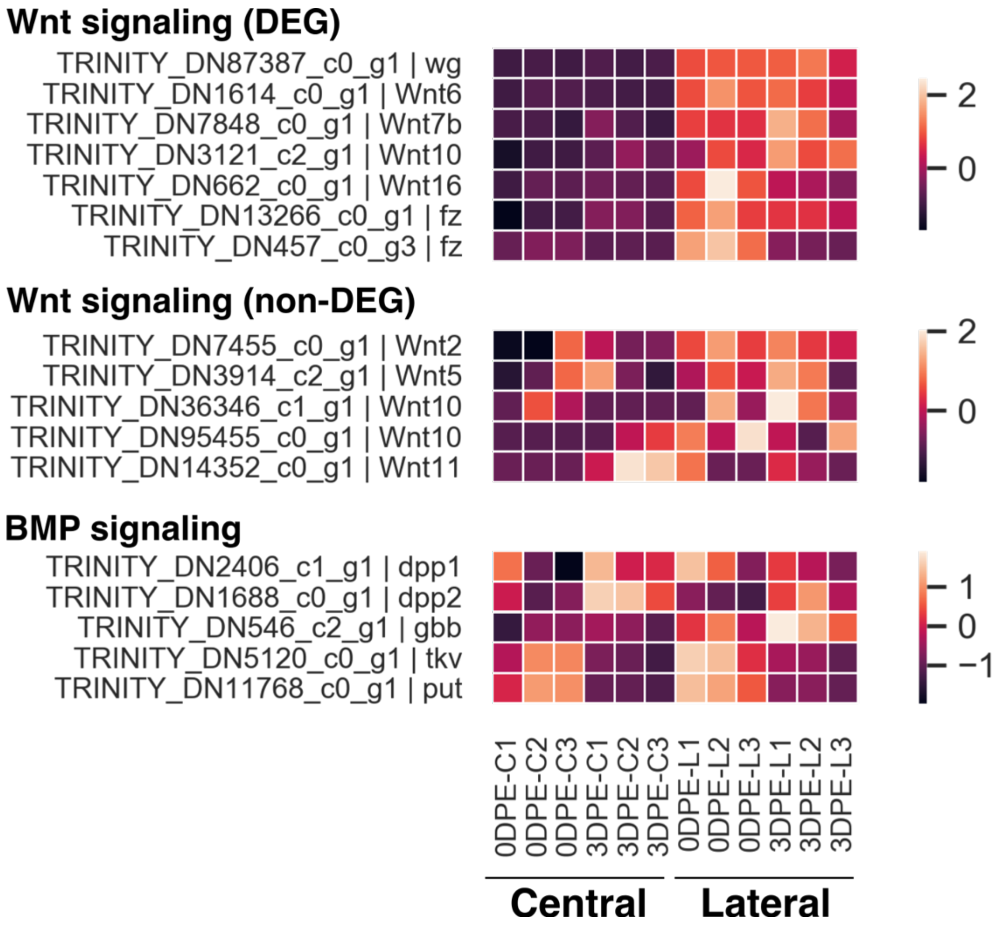

Supplementary Figure 10 Expression profile of components of Wnt and BMP signaling

Color code indicates z-score normalized TPM.

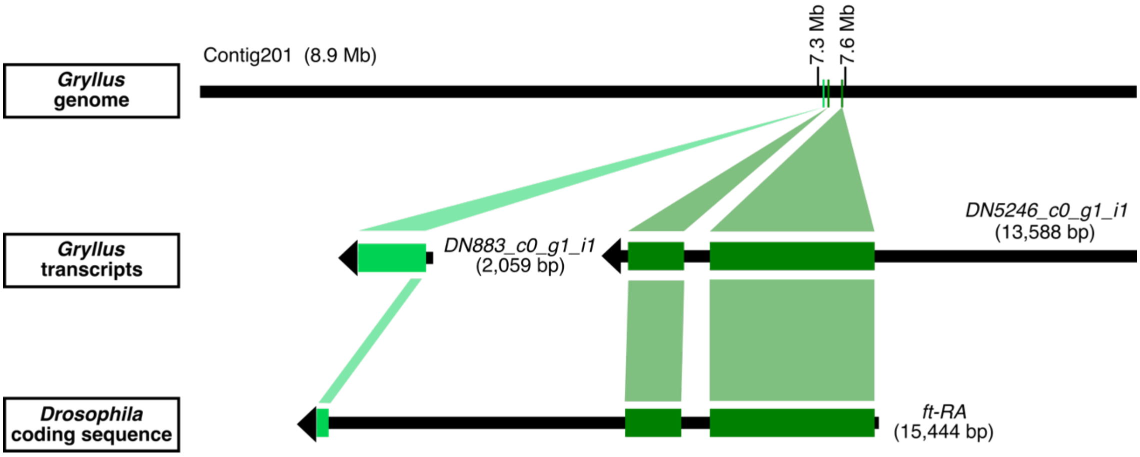

Supplementary Figure 11 Two *ft* transcripts of *Gryllus* derive from a single genomic locus
The contig201 in the *Gryllus* genome, and two transcripts of *ft* in the *Gryllus* transcriptome and the protein-coding sequence of a *ft* transcript in *Drosophila* are drawn with a black bar and black arrows, respectively. Color boxes indicate the corresponding regions among these nucleotide sequences according to a BLAST analysis. Two *ft* transcripts of *Gryllus* hit to the single *Drosophila ft* gene, and to the genomic locus within 300 kb. The order of the blast-hit sites on the genome agrees that two transcripts derive from a single *ft* gene.

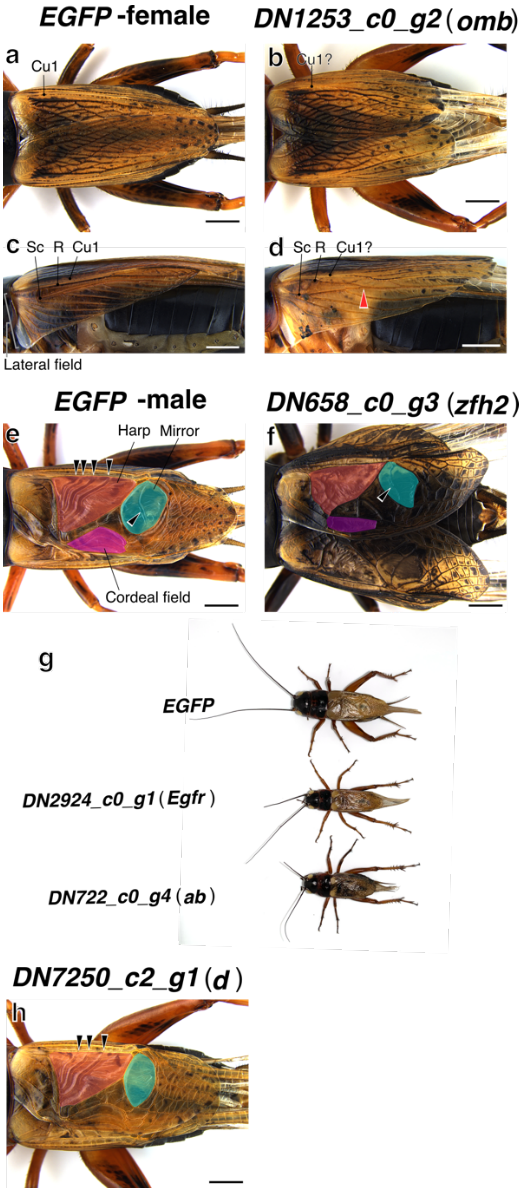

Supplementary Figure 12 RNAi phenotypes of *omb*, *zfh2*, *Egfr*, *ab* and *d*

a–**d** Dorsal (**a**, **b**) and lateral (**c**, **d**) views of female adults after *EGFP* (**a**, **c**) and *omb* (**b**, **d**) dsRNA injections. Characteristic veins were annotated following Schaffner & Koch (1987)[2]. Cu1, cubitus 1; R, radius; Sc, subcostal. R and Sc are fused (arrowhead) in the *omb* RNAi cricket but not in control. **e**, **f** Dorsal views of male adults after *EGFP* (**e**) and *zfh2* (**f**) dsRNA injection. The *zfh2* RNAi cricket displays abnormal wing vein patterns represented as a loss of organized cross-veins in both the harp (red) and cordeal fields (magenta), and the deformed mirror (cyan). **g** Adult male crickets after *EGFP*, *Egfr* and *ab* dsRNA injection at the same magnification. **h** Dorsal view of adult males after *d* dsRNA injection showing loss of cross-veins in the harp and mirror. Mirror also shows a deformed shape compared to control (**e**). Scale bar is 2 mm.

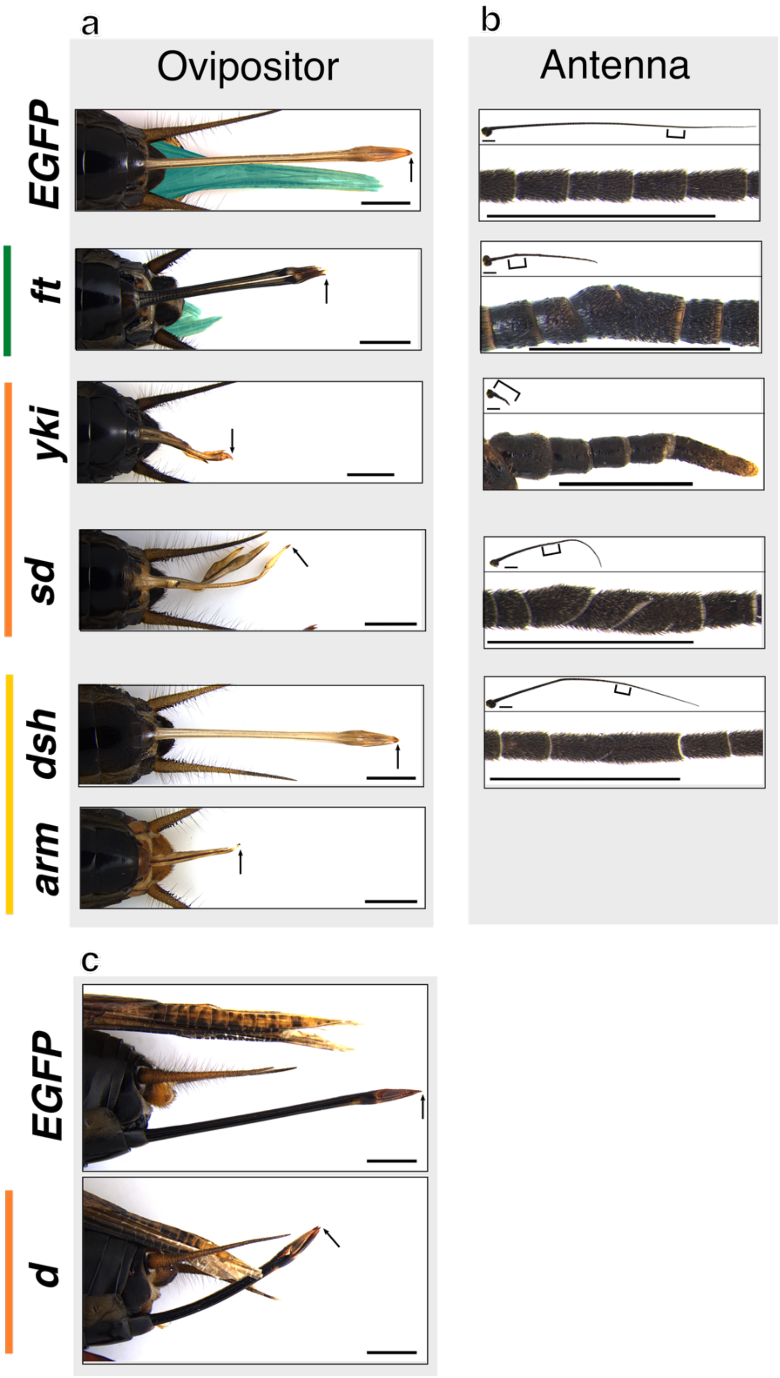

Supplementary Figure 13 RNAi effects of Wnt/Ft-Ds/Hippo pathway components on ovipositors and antennae

a Ventral view of posterior-end regions of RNAi adult crickets. The hindwing is shaded in cyan. Arrow indicates a distal end of an ovipositor. Scale bar is 1 mm. **b** Lateral views of posterior-end regions of *EGFP* and *d* RNAi crickets. **c** Bracketed part of the whole antenna (top) is magnified in the bottom panel. Scale bars are 1 mm.

Supplementary Table 1 Summary of mosaic knockouts

sgRNA	No. of injected egg	No. of hatched nymph (%)	No. of embryo died just before hatching (%)	Phenotype (%)		
				No effect	Moderate	Strong
EGFP	213	65 (30.5)	2 (0.93)	67 (100)	0 (0)	0 (0)
apAB	215	19 (8.84)	102 (47.4)	5 (4.13)	37 (30.6)	79 (65.3)
vg	212	45 (21.2)	18 (8.49)	38 (60.3)	24 (38.1)	1 (1.59)

Supplementary Table 2 Summary of nRNAi-mediated functional screening

Target	FlyBase FBgn	Gene symbol	ug injected dsRNA / nymph	No. injected	No. of male adult	No. of female adult	%survival rate	Adult phenotype				Note
								Small wing size	Other wing phenotype	Other specific phenotype	wildtype	
EGFP	-	-	5	13	8	4	92.3	0	0	0	12	
TRINITY_DN1122_c0_g3	FBgn0005771	noc	6.28	13	2	0	15.4	0	0	0	2	
TRINITY_DN1253_c0_g2	FBgn0000179	bi (omb)	6.69	13	3	3	46.2	0	6	0	0	
TRINITY_DN13266_c0_g1	FBgn0001085	fz	7.60	14	2	8	71.4	0	0	0	10	
TRINITY_DN14433_c0_g1	FBgn0037384	dgrn	7.33	12	10	1	91.7	0	0	0	11	
TRINITY_DN1533_c0_g1	FBgn0003892	ptc	6.75	13	4	6	76.9	0	0	0	10	
TRINITY_DN1540_c2_g1	No BLAST hit	Smbt2	6.79	13	7	3	76.9	0	0	0	10	
TRINITY_DN1614_c0_g1	FBgn0031902	Wnt6	6.45	13	5	4	69.2	0	0	0	9	
TRINITY_DN177_c0_g3	FBgn0001075	ft	5.38	8	3	1	50.0	0	0	0	4	
TRINITY_DN2297_c0_g2	FBgn0000658	fj	7.14	12	7	2	75.0	0	0	0	9	
TRINITY_DN263_c0_g2	FBgn0263118	tx	6.09	14	6	5	78.6	0	0	0	11	
TRINITY_DN2910_c3_g1	FBgn0004583	ex	6.71	14	5	5	71.4	0	0	0	10	
TRINITY_DN2924_c0_g1	FBgn0003731	Egfr	7.09	12	3	4	58.3	0	0	7	0	7x Small body size
TRINITY_DN3121_c2_g1	FBgn0031903	Wnt10	7.43	13	3	5	61.5	0	0	0	8	
TRINITY_DN3526_c3_g1	FBgn0085424	nub	7.25	13	0	2	15.4	0	0	0	2	
TRINITY_DN411_c6_g1	FBgn0036494	Toll-6	6.58	9	1	2	33.3	0	0	0	3	
TRINITY_DN411_c6_g2	FBgn0029114	Tollo	6.83	11	5	5	90.9	0	0	0	10	
TRINITY_DN457_c0_g3	FBgn0001085	fz	5.41	13	6	4	76.9	0	0	0	10	
TRINITY_DN5246_c0_g1	FBgn0001075	ft	6.26	13	8	0	61.5	7	0	0	1	
TRINITY_DN5740_c0_g1	FBgn0044028	Notum	7.22	14	7	1	57.1	0	0	0	8	
TRINITY_DN5792_c0_g3	FBgn0003450	snk	7.33	14	6	1	50.0	0	0	0	7	
TRINITY_DN658_c0_g3	FBgn0004607	zfh2	6.08	13	3	0	23.1	0	3	0	0	3x irregular vein pattern; 2x failed to eclose completely
TRINITY_DN662_c0_g1	FBgn0004360	Wnt16	7.43	14	4	3	50.0	0	0	0	7	
TRINITY_DN695_c0_g2	FBgn0004839	otk	6.14	14	2	5	50.0	0	0	0	7	
TRINITY_DN722_c0_g4	FBgn0264442	ab	6.16	13	8	2	76.9	0	0	8	2	8x small body size
TRINITY_DN7250_c2_g1	FBgn0262029	d	5.76	14	8	4	85.7	0	6	4	2	6x defects in male vein pattern (the AP vein in the mirror); 4x short and curved ovipositors
TRINITY_DN7341_c0_g2	FBgn0000492	Dr	6.36	13	4	2	46.2	0	0	0	6	
TRINITY_DN7377_c0_g1	FBgn0004644	hh	6.38	14	4	5	64.3	0	0	0	9	
TRINITY_DN7848_c0_g1	FBgn0004360	Wnt2	5.28	14	4	3	50.0	0	0	0	7	
TRINITY_DN8291_c0_g1	FBgn0039286	dan	1.54	14	6	4	71.4	0	0	0	10	
TRINITY_DN87387_c0_g1	FBgn0284084	wg	5.79	14	6	1	50.0	0	0	0	7	
TRINITY_DN876_c5_g2	FBgn0034476	Toll-7	5.10	14	4	6	71.4	0	0	0	10	
TRINITY_DN883_c0_g1	FBgn0001075	ft	5.52	14	6	6	85.7	12	0	0	0	
TRINITY_DN9479_c0_g1	FBgn0052405	Cpr65Av	5.32	14	6	2	57.1	0	0	0	8	

Supplementary Table 3 Summary of additional nRNAi analysis

Target	Amount of injected dsRNA / nymph	Injected instar	No. injected	No. of male adult	No. of female adult	%survival rate	No. of small winged adult (%)	Note
Experiment #1								
EGFP	3 μg	3rd	34	10	15	73.5	0 (0)	
disheveled (*dsh*)	3 μg	3rd	29	10	13	79.3	23 (100)	
yorkie (*yki*)	3 ng	3rd	6	0	0	0.0	*N.A.*	
scalloped (*sd*)	3 μg	3rd	35	10	11	60.0	21 (100)	Other phenotype: 21 x short antennae; 11 x short ovipositors
Experiment #2								
EGFP	50 ng	6th	23	5	7	52.2	0 (0)	
yorkie (*yki*)	30 ng	6th	22	0	0	0.0	*N.A.*	
yorkie (*yki*)	3 ng	6th	24	8	8	66.7	16 (100)	Other phenotype: 16 x short antennae and ovipositors
Experiment #3								
EGFP	5 μg	3rd	15	8	3	73.3	0 (0)	
armadillo (*arm*)	10 ng	3rd	17	0	0	0.0	*N.A.*	
armadillo (*arm*)	1 ng	3rd	18	2	2	22.2	4 (10)	Other phenotype: 2 x short ovipositor, 3 x short antennae, 1 x small body size
Experiment #4								
EGFP	5 μg	3rd	13	0	7	53.8	0 (0)	
armadillo (*arm*)	1 ng	3rd	24	3	6	37.5	8 (88.8)	Other phenotype: 8 x short antennae and ovipositors and small body size

Supplementary Table 4 Oligonucleotides used

Target	Purpose	Sequence #1	Sequence #2	bp product size
in situ hybridization				
wingless (*wg*)	PCR primer	atttaggtgacactatagaaCTCTGCGGGAGAAGATGAAC	taatacgactcactatagggCACGTCTTGCACTTGACCTC	523
apterous AB (*apAB*)	PCR primer	atttaggtgacactatagaaATCTGAACGCGGACTACCTG	taatacgactcactataggGTCTTCTGCGAGAGCTGCTT	223
vestigial (*vg*)	PCR primer	atttaggtgacactatagaaCAGTACGTCTCCGCCAACTG	taatacgactcactataggGCCTGGTAGTTGCTGTTCCA	188
Generation of *vg* reporter line				
Oligonucleotides for sgRNA synthesis				
vg 5′ region	sgRNA template	taatacgactcactatagACTGATCGCCCTCGA	ttctagctctaaaacGGCACTCGAGGGCGATCAG	*N.A.*
G'act-eGFP donor plasmid	sgRNA template	taatacgactcactatagGATGTCCCAGGCGAA	ttctagctctaaaacGCCCTTCGCCTGGGACATC	*N.A.*
Primers for genomic PCR				
Junction sequence between genome and the donor	PCR primer	TTCCTCGACGAGTGTGTCTG	GCTTCGAAAAGTGTCGGCTA	354
Expression of ecdysone responsive genes				
E74	qPCR primer	ATGCTCGACCTGGGCTTCCA	AGAACCTTCGCGGCTCTTGC	108
E75	qPCR primer	CGCCTCGCCTGTATGTTCGA	GCGTTCGACGACGAGTGGAT	95
Ribosomal protein L32 (*RpL32*)	qPCR primer	GATTCGCCCAGTTTATCGTC	GGCTTCAGCTTCTTGTACCG	90
nRNAi-mediated functional screening				
TRINITY_DN1122_c0_g3 (noc)	PCR primer	taatacgactcactataggGACGACTTGGAGGGCTTCTC	taatacgactcactataggGAACACTGAACCGGGAGTGG	131
TRINITY_DN1253_c0_g2 (bi)	PCR primer	taatacgactcactataggGCGAGTCAGGGTGGATGTAC	taatacgactcactatagggTCCCGCAGATGAAATTCCGT	204
TRINITY_DN14433_c0_g1 (dgrn)	PCR primer	taatacgactcactatagggTTTCCTAGCACGATGGCTCC	taatacgactcactatagggCTGAGGGACTGTTGAGGTGG	200
TRINITY_DN1533_c0_g1 (ptc)	PCR primer	taatacgactcactataggGAATTGGTGGTGGCTGCAAG	taatacgactcactatagggACATGTGCATGGCAGGAAGA	199
TRINITY_DN1540_c2_g1 (Smbt2)	PCR primer	taatacgactcactatagggACGGCTGGCAGTGATTAAGT	taatacgactcactatagggTGGCTTCACTGCTACCCAAG	203
TRINITY_DN1614_c0_g1 (Wnt6)	PCR primer	taatacgactcactatagggTCGACCTGTCTTGTGCACTC	taatacgactcactataggGCAAGGTCAGCGTCAAAGTG	200
TRINITY_DN177_c0_g3 (ft)	PCR primer	taatacgactcactataggGAGATCGAGCGCCTCAACTC	taatacgactcactataggGAGCTGTGGTTGTCCAGGTG	215
TRINITY_DN2297_c0_g2 (fj)	PCR primer	taatacgactcactataggGAATCGCAATGAGCACGGTC	taatacgactcactataggGCGGCTGCATATAAATCGCC	200
TRINITY_DN263_c0_g2 (tx)	PCR primer	taatacgactcactataggGCTGTCACGAGAAGAGGTCC	taatacgactcactatagggATGTCTCCGCACGACTTGTT	199
TRINITY_DN2910_c3_g1 (ex)	PCR primer	taatacgactcactatagggAGCATTGTACGGTGGTGGAG	taatacgactcactataggGCGCAACCATTAGCACTACG	200
TRINITY_DN2924_c0_g1 (Egfr)	PCR primer	taatacgactcactatagggTTCACAATGCCAGAACGGGA	taatacgactcactatagggTGTTTAGGACCACGACAGCC	197
TRINITY_DN3121_c2_g1 (Wnt10)	PCR primer	taatacgactcactatagggTGCCATCGAGGAGTAGGTCA	taatacgactcactatagggCATCTTCTGCCGCATTGACG	209
TRINITY_DN3526_c3_g1 (nub)	PCR primer	taatacgactcactatagggCCCAAGAACAGGCTGAGGAG	taatacgactcactataggGCAAAGTCACAACAGCCCTG	126
TRINITY_DN411_c6_g1 (Toll-6)	PCR primer	taatacgactcactatagggTCGCACTCGACAAACAGTTC	taatacgactcactatagggCTTCAACCACTTCGTGCTGA	181
TRINITY_DN411_c6_g2 (Tollo)	PCR primer	taatacgactcactatagggCAGTTGGTAGGGCACGTCAT	taatacgactcactatagggAGTGCTCGAAATCAGCCGAA	200
TRINITY_DN457_c0_g3 (fz)	PCR primer	taatacgactcactatagggAGACTTCGCTAAGGCACCTG	taatacgactcactataggGCCACGAATCCTTTCTGTGC	199
TRINITY_DN5246_c0_g1 (ft)	PCR primer	taatacgactcactatagggAACGACAACCCGCCCATATT	taatacgactcactatagggCGGCTCGGTGGTAGAAATGT	201
TRINITY_DN5740_c0_g1 (Notum)	PCR primer	taatacgactcactatagggAGACGAGCTACGCGAGAGTA	taatacgactcactatagggCTTTGGTTGCACGCCCAAAT	223
TRINITY_DN5792_c0_g3 (snk)	PCR primer	taatacgactcactataggGACTTCAGTCGGCCTTGTGA	taatacgactcactatagGGGATGGTACTTTCACTGCCA	195
TRINITY_DN658_c0_g3 (zfh2)	PCR primer	taatacgactcactatagggTGCGCTCTGTACTGCATCAA	taatacgactcactatagggTGCCACAACTACTGTTCGCT	201
TRINITY_DN662_c0_g1 (Wnt16)	PCR primer	taatacgactcactatagggTGGATGTGCACTAGATCGCC	taatacgactcactatagggTCGATGCCATGGAATCTCCG	198
TRINITY_DN695_c0_g2 (otk)	PCR primer	taatacgactcactatagggAAGACGGTGATCCTGGAGGT	taatacgactcactatagggCCACCAACTCCTCCAGGAAC	239
TRINITY_DN722_c0_g4 (ab)	PCR primer	taatacgactcactataggGGAAGGCACTCCCTCACTTC	taatacgactcactatagGGGTGATTGTCGTCCATCGT	201
TRINITY_DN7250_c2_g1 (d)	PCR primer	taatacgactcactatagggCGGCAATACAGAGCTTTCGC	taatacgactcactatagggTGGTCAGGGACTAGAGGTGG	201
TRINITY_DN7341_c0_g2 (Dr)	PCR primer	taatacgactcactataggGCAAGAGTCATACACGCACG	taatacgactcactatagggTGCTTCGGAAACAATGTGCG	200
TRINITY_DN7377_c0_g1 (hh)	PCR primer	taatacgactcactatagggATGCTCTCTCTCACTGTGCG	taatacgactcactatagggAGGCCCAGCTTTGAACACTT	200
TRINITY_DN7848_c0_g1 (Wnt2)	PCR primer	taatacgactcactataggGCATGCTCACACACATCCAC	taatacgactcactatagggAGTCGAGCAATACACGTGCA	176
TRINITY_DN8291_c0_g1 (dan)	PCR primer	taatacgactcactatagggACCTGGTCTACGTGCCATTG	taatacgactcactatagggAGCTCTGCACAGACAATGCT	122
TRINITY_DN87387_c0_g1 (wg)	PCR primer	taatacgactcactataggGAAGCACTGTACGGCTGGAA	taatacgactcactatagggAGTTCACTGTCCGCCGAAAT	196
TRINITY_DN876_c5_g2 (Toll-7)	PCR primer	taatacgactcactatagggAGCTTCTCGTGCTCGATCTG	taatacgactcactataggGTCCTCGATGTAGCCGATGG	200
TRINITY_DN883_c0_g1 (ft)	PCR primer	taatacgactcactatagggACAAGCAACCAGTCCTCAGG	taatacgactcactatagggCCAAGCCCTCCGAAGAGTAC	191
TRINITY_DN9479_c0_g1 (Cpr65Av)	PCR primer	taatacgactcactatagggCCCCGTTTTCGGGTATAAAT	taatacgactcactatagggAGAGCTGCCGAGGAGATGT	125
Additional nRNAi				
disheveled (*dsh*)	PCR primer	taatacgactcactatagggCACGCACATCTTCCTTCTCA	taatacgactcactatagGGCTCTATGCGACCATCAAT	196
armadillo (*arm*)	PCR primer	taatacgactcactatagggATCCAAGTCAAAGGCTGGTG	taatacgactcactataggGCGCTGATTGTTGCAAGTTA	185
yorkie (*yki*)	PCR primer	taatacgactcactatagggCCCTGCAGATGGAGAGAGAG	taatacgactcactatagggTGATCAGTCACGCCTGAGA	143
scalloped (*sd*)	PCR primer	taatacgactcactatagggCCAGGTGTTGGCTAGAAGGA	taatacgactcactatagggCCTGGGTAGGACACTGGAGA	207

Small letters indicate universal sequences

Supplementary Reference

1. Furukawa, N., Tomioka, K. & Yamaguchi, T. Functional anatomy of the musculature and innervation of the neck and thorax in the cricket, *Gryllus bimaculatus*. *Zool. Mag.* **92**, 371–385 (1983).
2. Schaffner, K. H. & Koch, U. T. A new field of wing campaniform sensilla essential for the production of the attractive calling song in crickets. *J. Exp. Biol.* **129**, 1–23 (1987).
