# Supplementary material for: A hemimetabolous wing development suggests the wing origin from lateral tergum of a wingless ancestor
Source: Nat Commun. 2022 Feb 21;13:979. doi: 10.1038/s41467-022-28624-x (PMC8861169; doi:10.1038/s41467-022-28624-x)
Supplement: Supplementary file 3 — Reporting Summary [file 41467_2022_28624_MOESM3_ESM.pdf]

nature portfolio

Corresponding author(s): Takahiro Ohde

Last updated by author(s): Dec 6, 2021

Reporting Summary

Nature Portfolio wishes to improve the reproducibility of the work that we publish. This form provides structure for consistency and transparency in reporting. For further information on Nature Portfolio policies, see our Editorial Policies and the Editorial Policy Checklist.

Statistics

For all statistical analyses, confirm that the following items are present in the figure legend, table legend, main text, or Methods section.

n/a	Confirmed	
☐	☒	The exact sample size (*n*) for each experimental group/condition, given as a discrete number and unit of measurement
☐	☒	A statement on whether measurements were taken from distinct samples or whether the same sample was measured repeatedly
☐	☒	The statistical test(s) used AND whether they are one- or two-sided<br>*Only common tests should be described solely by name; describe more complex techniques in the Methods section.*
☒	☐	A description of all covariates tested
☒	☐	A description of any assumptions or corrections, such as tests of normality and adjustment for multiple comparisons
☐	☒	A full description of the statistical parameters including central tendency (e.g. means) or other basic estimates (e.g. regression coefficient) AND variation (e.g. standard deviation) or associated estimates of uncertainty (e.g. confidence intervals)
☐	☒	For null hypothesis testing, the test statistic (e.g. *F*, *t*, *r*) with confidence intervals, effect sizes, degrees of freedom and *P* value noted<br>*Give P values as exact values whenever suitable.*
☒	☐	For Bayesian analysis, information on the choice of priors and Markov chain Monte Carlo settings
☒	☐	For hierarchical and complex designs, identification of the appropriate level for tests and full reporting of outcomes
☒	☐	Estimates of effect sizes (e.g. Cohen's *d*, Pearson's *r*), indicating how they were calculated

Our web collection on statistics for biologists contains articles on many of the points above.

Software and code

Policy information about availability of computer code

Data collection	Leica M165 FC and A1R MP are controlled by LAS X (v3.4.1) and NIS-Elements AR (v4.13) software, respectively.
Data analysis	Softwares below are used for the RNA-seq analysis:<br>Cutadapt (v2.9)—read trimming, Trinity (v2.8.4)—short read assembly, BUSCO (v4.0.5)—quality evaluation of an assembly, Salmon (v1.0.1)—transcript quantification, DESeq2 (v1.26.0)—differential expression analysis, Trinotate (v3.1.0)—assembly annotation, BLASTX (v2.9.0)—assembly annotation, DAVID (v6.8)—gene function analysis<br>Software below is used for image analysis:<br>Fiji (ImageJ2, v2.0.0)<br>Softwares below are used for data visualization:<br>DABEST (v0.3.1), EnhancedVolcano (v1.7.10), Python3 (v3.7.7), R (v3.6.3), GIMP (v2.10), Inkscape (v1.0 beta)

For manuscripts utilizing custom algorithms or software that are central to the research but not yet described in published literature, software must be made available to editors and reviewers. We strongly encourage code deposition in a community repository (e.g. GitHub). See the Nature Portfolio guidelines for submitting code & software for further information.

Data

Policy information about availability of data

All manuscripts must include a data availability statement. This statement should provide the following information, where applicable:

- Accession codes, unique identifiers, or web links for publicly available datasets
- A description of any restrictions on data availability
- For clinical datasets or third party data, please ensure that the statement adheres to our policy

Nucleotide sequences of Gryllus orthologs and the RNA-seq data characterized and generated in this study are deposited to DDBJ/EBI/NCBI database under accession numbers LC589559 (vg), LC589561 (apA), LC589562 (apB) and PRJDB10701, respectively. Raw data for area measurement and ablation experiment are provided in Source Data.

Field-specific reporting

Please select the one below that is the best fit for your research. If you are not sure, read the appropriate sections before making your selection.

- [x] Life sciences
- [ ] Behavioural & social sciences
- [ ] Ecological, evolutionary & environmental sciences

For a reference copy of the document with all sections, see nature.com/documents/nr-reporting-summary-flat.pdf

Life sciences study design

All studies must disclose on these points even when the disclosure is negative.

Sample size	No statistical method was used to determine sample sizes in this study. Sample sizes were determined by considering the variability of results, and the cost and time for obtaining the data. We predicted the variability of results from previous experiences with similar experiments or from pilot experiments. For RNA-seq analysis, the cost was the major determinant of the sample size (n=3).
Data exclusions	No data were excluded from the analyses.
Replication	For gene expression analysis the same experiments were repeated at least three times and representative results displayed in the article were confirmed. For functional analyses by using CRISPR/Cas and RNAi, the number of biological replicates and results are shown in Supplementary Table 1–3. For the ablation experiment, each treatment was repeated 6 to 17 times, and consistent results were confirmed as shown in Fig. 3. For the RNA-seq analysis, three biological replicates were sequenced to take into account the variation, and overall similarities among replicates were confirmed with the distance matrix shown in Supplementary Fig. 8a. For documenting the vg reporter gene expression, at least three independent specimens of embryo or nymph were observed, and the similar expression patterns were confirmed.
Randomization	Biological samples used in this study are randomized because cricket samples for each experiment were randomly picked from laboratory cultures.
Blinding	The investigator was not blinded during data collection and analysis in this study. For gene expression and functional analyses, randomly picked samples were treated equally for each target gene according to the same protocol. For image and RNA-seq analyses, each sample was analyzed according to the same computational pipeline.

Reporting for specific materials, systems and methods

We require information from authors about some types of materials, experimental systems and methods used in many studies. Here, indicate whether each material, system or method listed is relevant to your study. If you are not sure if a list item applies to your research, read the appropriate section before selecting a response.

Materials & experimental systems

n/a	Involved in the study	
[ ]	[x]	Antibodies
[x]	[ ]	Eukaryotic cell lines
[x]	[ ]	Palaeontology and archaeology
[ ]	[x]	Animals and other organisms
[x]	[ ]	Human research participants
[x]	[ ]	Clinical data
[x]	[ ]	Dual use research of concern

Methods

n/a	Involved in the study	
[x]	[ ]	ChIP-seq
[x]	[ ]	Flow cytometry
[x]	[ ]	MRI-based neuroimaging

Antibodies

Antibodies used	Anti-Digoxigenin-AP, Fab fragments; Roche, 11093274910

Validation	Validation statements are on the manufacturer's website: https://www.sigmaaldrich.com/US/en/product/roche/11093274910

Animals and other organisms

Policy information about studies involving animals; ARRIVE guidelines recommended for reporting animal research

Laboratory animals	We used Gryllus bimaculatus gwhite strain from Tokushima University and vg5GFP strain generated in this study. Both male and female, and animals from embryo to adult stages were used for this study.
Wild animals	This study did not involve wild animals.
Field-collected samples	This study did not involve samples collected from the field.
Ethics oversight	No ethical approval or guidance was required for insects in the institutes where this study is done.

Note that full information on the approval of the study protocol must also be provided in the manuscript.
